# HCV Diversity among Chinese and Burmese IDUs in Dehong, Yunnan, China

**DOI:** 10.1371/journal.pone.0163062

**Published:** 2016-09-22

**Authors:** Zhenzhou Wan, Qianqian Chen, Xin Chen, Lin Duo, Peilu Li, Yong-Tang Zheng, Chiyu Zhang

**Affiliations:** 1 Pathogen Diagnostic Center, CAS Key Laboratory of Molecular Virology & Immunology, Institut Pasteur of Shanghai, Chinese Academy of Sciences, Shanghai 200031, China; 2 Key Laboratory of Animal Models and Human Disease Mechanisms of Chinese Academy of Sciences & Yunnan Province, Kunming Institute of Zoology, Chinese Academy of Sciences, Kunming 650223, China; 3 Medical Laboratory of Taizhou Fourth People’s Hospital, Taizhou, Jiangsu 225300, China; 4 The Second People’s Hospital of Yunnan Province, Kunming, Yunnan 650031, China; 5 Kunming College of Life Science, University of Chinese Academy of Sciences, Kunming, Yunnan 650204, China; Fudan University, CHINA

## Abstract

HCV transmission is closely associated with drug-trafficking routes in China. Dehong, a prefecture of Yunnan, is the important trade transfer station linking Southeast Asia and China, as well as the drug-trafficking channel linking “Golden triangle” and other regions of China and surrounding countries. In this study, we investigated the HCV genotype diversity among IDUs in Dehong based on 259 HCV positive samples from 118 Chinese and 141 Burmese IDUs. HCV genotypes were determined based on the phylogenies of C/E2 and NS5B genomic sequences. Six HCV subtypes, including 1a, 1b, 3a, 3b, 6n and 6u, were detected. Interestingly, 4 HCV sequences from Burmese IDUs did not cluster with any known HCV subtypes, but formed a well-supported independent clade in the phylogenetic trees of both C/E2 and NS5B, suggesting a potential new HCV subtype circulating in Dehong. Subtype 3b was the predominant subtype, followed by subtypes 6n and 6u. Comparison showed that Dehong had a unique pattern of HCV subtype distribution, obviously different from other regions of China. In particular, HCV subtypes 6u and the potential new HCV subtype had a relatively high prevalence in Dehong, but were rarely detected in other regions of China. There was no significant difference in HCV subtype distribution between Burmese and Chinese IDUs. Few HCV sequences from Burmese and Chinese IDUs clustered together to form transmission clusters. Furthermore, about half of HCV sequences from Burmese IDUs formed small transmission clusters, significantly higher than that from Chinese IDUs (*p*<0.01). These suggest that the Chinese and Burmese IDUs were relatively isolated from each other in injection drug use behavior and the Burmese IDUs might prefer to inject drugs themselves together. The unique genotype distribution and complex diversity of genotype 6 among IDUs may be associated with the special geographical position of Dehong.

## Introduction

Hepatitis C virus (HCV) is an enveloped virus with a positive-sense single-stranded RNA. HCV belongs to the genus *Hepacivirus*, a member of Flaviviridae family, and is a main cause of chronic liver disease. It often leads to liver cirrhosis or hepatocellular carcinoma [1]. HCV transmission is predominantly through intravenous drug use (IDU), blood transfusion and sexual contact [2]. In China, high proportions (15.6%-98.7%) of intravenous drug users (IDUs) were detected to be HCV positive, indicating that IDU has become the most predominant mode of HCV transmission [3]. HCV has been classified into at least 6 major genotypes and each genotype is further divided into some subtypes [4]. The genotypes 1, 2 and 3 are globally epidemic, while others are generally confined to specific geographical regions [5]. The genotypes of HCV were demonstrated to associate with the response to antiviral therapy [6,7]. In China, at least 4 genotypes were prevalent. Subtype 1b was the most common subtype among general population and subtypes 3a, 3b, and 6a were often circulating among IDUs [8,9].

**Fig 1:**
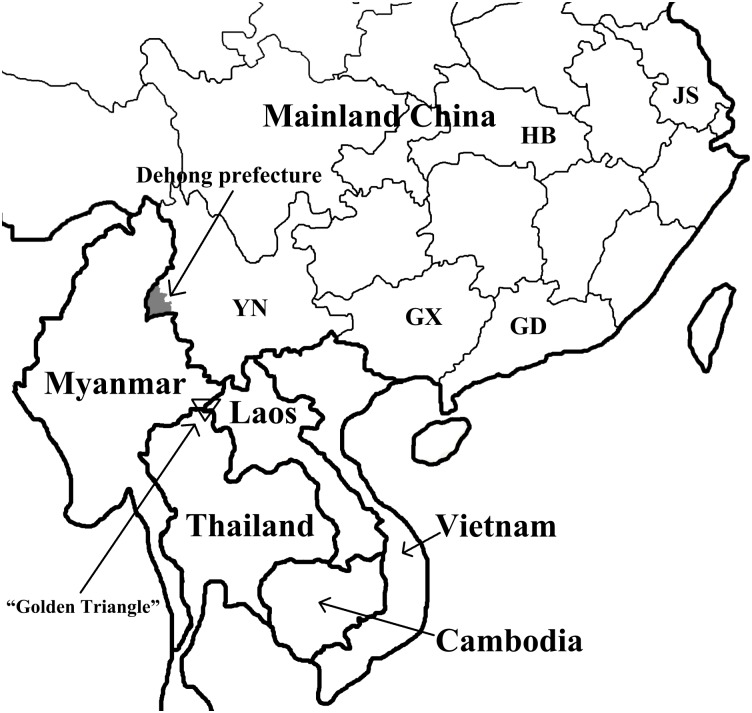
The geographical location of Dehong, Yunnan, China. YN: Yunnan; GX: Guangxi; GD: Guangdong; HB: Hubei; JS: Jiangsu.

HCV transmission was closely with drug-trafficking routes and migration of population especially in southwestern region of China [10–12]. Dehong Dai-Jingpo autonomous prefecture is located in southwestern China. It shares a long border with Myanmar. Travel, trade and intermarriage are very common in this region. Dehong is a key channel for illegal drug trafficking from “Golden triangle”, which is comprised of large regions of Myanmar, Laos and Thailand, to China and other countries/regions (Fig 1). In recent years, more and more Burmese select to work, live, and consume in Dehong, Yunnan, China, due to local rapid economic development and increasing economic communication between China and Myanmar. Most of them return to Myanmar several times each year. Furthermore, some Burmese and Chinese IDUs often crossed China-Myanmar border to inject drugs, which objectively increases the transmission of many infectious diseases (e.g. HCV and HIV-1) in this area [11]. The special geographical location and convenient China-Myanmar border port provide more chances for HCV transmission among IDUs in Dehong, thereby resulting in more complex HCV diversity.

In this study, we investigated the HCV genotypes and subtypes distribution among IDUs (including Burmese and Chinese) in Dehong. We defined a potential novel HCV subtype, which forms a well supported cluster between 6m and 6n clades in phylogeny, and found that Dehong had a unique pattern of HCV genotype distribution with high prevalence rate of 6u. In addition, we found that almost half of HCV sequences from Burmese IDUs formed small transmission clusters, significantly higher than that from Chinese IDUs.

## Results

### Social-demographic Characterization of HCV infected IDUs in Dehong, Yunnan, China

The social-demographic information of HCV infected IDUs in Dehong, China, including occupation, ethnic, marital status, educational level, and age, is listed in S1 Table. The individuals without available information were excluded from the statistics. Majority of these IDUs were farmer (88.2%), and 26–40 years old (72.7%), and had completed their primary school or higher level education (82.8%). The single and married IDUs had similar proportions, accounting for 45.8% and 40.7%, respectively. Dai, Han and Jingpo were the top three common ethnic groups in this cohort, accounting for 31.5%, 31.1%, and 26.7%, respectively. Chinese and Burmese IDUs showed significant differences in ethnic background and marriage status (*p*<0.05). In ethnic background, Han (42.9%) and Dai (40.2%) are the top two common ethnics in Chinese IDUs, while Jingpo (38.1%) and Dai (24.5%) are the top two common ethnics in Burmese IDUs. In marriage status, more than half (51.1%) of Burmese IDUs were married, higher than the number (28.3%) in Chinese IDUs. In contrast, the proportion of unmarried cohabitation was 2.2% for Burmese IDUs, lower than that (16.8%) for Chinese IDUs. There is no significant difference in occupation, education status and age between Chinese and Burmese IDUs (*p*>0.05).

### HCV genotyping of the sequences from IDUs in Dehong, Yunnan, China

From 259 HCV positive samples, we successfully amplified and sequenced 175 C/E2 and 147 NS5B fragments. There are 138 samples having both NS5B and C/E2 sequences, and 46 samples having either NS5B or C/E2 sequences. The sequences were deposited in GenBank under accession numbers KM284803-KM284977 for C/E2 and KM284978-KM285124 for NS5B. Some samples were failed in the amplification of HCV genomic fragments might be due to primer specificity, low viral load and/or relatively low quality of samples caused by long-distance transportation and time delay.

**Fig 2:**
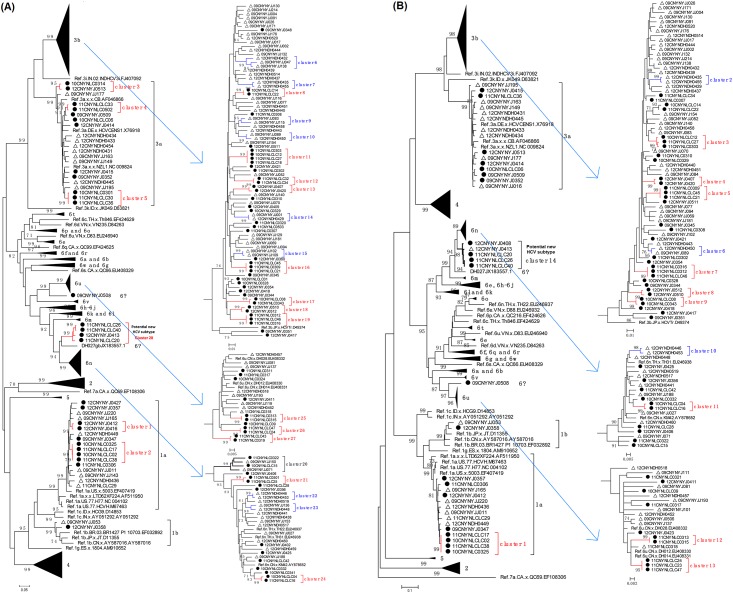
ML trees of C/E2 (A) and NS5B (B) fragments of HCV strains isolated from Dehong, China. The trees were constructed using MEGA 5.0 program with ML method under the “GTR+I+G” model. The reliability of both trees was evaluated by the bootstrap method with 1000 replications. Only bootstrap values > 70 are shown. Sequences from the Chinese and Burmese IDUs in Dehong are labeled by open triangle and solid circle, respectively. Transmission clusters formed by Chinese and Burmese IDUs are labeled in blue and red half-frames, respectively. If the transmission clusters contain both Chinese and Burmese IDUs, they are shown in black half-frame. The clade of the potential new HCV subtype is highlighted by a shadow.

To determine the genotypes of these samples, the phylogenetic trees of C/E2 and NS5B were constructed with the sequences in this study and the subtype references from HCV Database (Fig 2). In the C/E2 tree, all sequences from this study except 5 sequences can cluster with the subtype reference sequences, form well supported clades, and be defined as corresponding genotypes. For NS5B sequences, we observed a similar result to that of C/E2 sequences. All except 6 sequences can cluster with the subtype reference sequences, form well supported clades, and be defined as corresponding genotypes. HCV genotypes of 184 samples were determined based on the phylogenetic analyses of single (46 samples) or two (138 samples) genomic fragments (Fig 2). Six HCV subtypes, including 1a, 1b, 3a, 3b, 6n and 6u, were identified. The subtype results were completely consistent between the C/E2 and NS5B sequences for 138 samples having both sequences (Fig 2), suggesting that there was no inter-subtype recombinant. In addition, the subtype proportions were similar between C/E2 and NS5B fragments (Table 1). Taking the results of both genomic sequences together, 17, 2, 21, 84, 29, and 25 sequences were identified as 1a, 1b, 3a, 3b, 6n, and 6u, respectively. Several (5 C/E2 and 6 NS5B) sequences that did not cluster with any subtype reference might represent new HCV subtypes (Fig 2). Subtype 3b was the most predominant HCV genotype among these IDUs in Dehong, accounting for 45.4% of all 185 strains, followed by subtypes 6n and 6u with percentages of 15.7% and 13.5%, respectively (Table 2). Because these samples were collected from Chinese and Burmese IDUs, we compared the subtype distributions between both populations. No significant difference was observed in HCV subtype distribution between Burmese and Chinese IDUs (*p*>0.05) (Table 2).

**Table 1:** The genotypes distribution of HCV among IDUs in Dehong, China.

Genomic fragment		1a	1b	3a	3b	6n	6u	Potential new HCV subtypes	Total
C/E2		17	2	21	82	29	19	5	175
NS5B		14	2	16	69	20	20	6	147
Total	C/E2 and/or NS5B	17	2	22	83	29	25	6	184
	Percentage (%)	9.2	1.1	12.0	45.1	15.8	13.6	3.3	100

**Table 2:** Percentage distributions of HCV subtypes among Chinese and Burmese IDUs in Dehong, China.

Nationality	1a	1b	3a	3b	6n	6u	Potential new HCV subtypes	Total
Chinese IDUs (n = 90)	7.8	1.1	11.1	50.0	16.7	12.2	1.1	100
Burmese IDUs (n = 95)	10.5	1.1	12.6	41.1	14.7	14.7	5.3	100

### Finding of a potential new HCV subtype among IDUs in Dehong, Yunnan, China

There are 4 samples from Burmese IDUs unable to be defined as certain subtype. They all clustered with the strains of genotype 6 in both the C/E2 and NS5B trees (Fig 2), indicating that they should belong to genotype 6. All of them formed a well-supported independent clade (bootstrap value: 99%) and further clustered with the 6n clade in both the C/E2 and NS5B trees, indicating a closer genetic relationship with subtype 6n. To confirm whether these strains belong a potential new HCV subtype, we calculated the mean genetic distance within and between several related subtypes (i.e., 6a, 6u, 6v, 6k, 6m, and 6n) in genotype 6 (Table 3). Compared with the selected several genotype 6 subtypes, these strains of potential new HCV subtype had the lowest mean genetic distance within group, regardless of in the C/E2 and NS5B genomic fragments, and had the lowest mean genetic distance to subtype 6n in both C/E2 (0.225) and NS5B (0.095) genomic fragments (Table 3), supporting the close genetic relationship with subtype 6n. Furthermore, the mean genetic distance (C/E2: 0.225 and NS5B: 0.095) between the potential new HCV subtype and 6n was higher than the mean genetic distances within each of both subtypes (potential new HCV subtype, C/E2: 0.040 and NS5B: 0.015; 6n, C/E2: 0.098 and NS5B: 0.053), supporting these strains being a potential new HCV subtype.

**Table 3:** Mean genetic distances within and between subtypes of genotype 6.

Subtypes	Mean genetic distance between subtypes (C/E2 / NS5B)						Mean genetic distance within subtype (C/E2 / NS5B)
	Potential new HCV subtype	6u	6a	6v	6k	6m	
Potential new HCV subtype							0.040/0.015
6u	0.360/0.232						0.055/0.020
6a	0.437/0.247	0.442/0.219					0.059/0.045
6v	0.354/0.246	0.404/0.205	0.444/0.242				0.048/0.023
6k	0.326/0.194	0.417/0.261	0.442/0.258	0.403/0.264			0.187/0.104
6m	0.300/0.146	0.412/0.251	0.445/0.265	0.409/0.237	0.342/0.200		0.065/0.024
6n	0.225/0.095	0.394/0.215	0.439/0.263	0.379/0.241	0.317/0.194	0.311/0.154	0.098/0.053

Beside 4 sequences from Burmese IDUs, one NS5B sequence from Chinese IDUs also clustered within the clade of the potential new HCV subtype (Fig 2b). To further confirm the potential new HCV subtype, we performed HCV BLAST using the sequences of the potential HCV new subtype as the query set. The top hit sequences generated by HCV BLAST were subjected to further phylogenetic analyses with all other HCV genotype 6 sequences obtained in this study. We found that at least six (6 NS5B and 7 C/E2 sequences) HCV sequences from a previous study conducted in Dehong were highly similar to the query sequences [13]. Phylogenetic analyses showed that they closely clustered with the potential new HCV subtype, and formed a large transmission cluster (11 sequences) with a 100% bootstrap value support, indicating a local prevalence of this new HCV subtype (S1 Fig).

For another strain (09CNYNYJ-0508), it clustered with the clade of 6u, but formed an independent branch in both the C/E2 and NS5B trees, possibly also implying a new subtype.

### Transmission cluster analyses

IDU is the major HCV transmission mode especially in southwestern China (e.g. Yunnan province) [11,12]. In China-Myanmar border area, many IDUs often inject drugs together, thereby largely increasing the chance of HCV transmission among small groups and possibility of forming some transmission clusters. So, we investigated the formation of transmission clusters in different subtypes and populations. Both ML and NJ trees show similar topologies. Slightly more clusters were identified in NJ tree than in ML trees. So, we counted the numbers of the transmission clusters based on ML trees. We found 28 and 14 transmission clusters in C/E2 and NS5B sequences from IDUs in Dehong, respectively (Fig 2). All transmission clusters were small clusters containing 2–5 sequences. In Burmese IDUs, 42.9–100% C/E2 sequences of various subtypes formed transmission clusters (Table 4). In total, 49 (53.3%) of 92 C/E2 sequences from Burmese IDUs formed 19.5 transmission clusters, which was about two times more than 8.5 transmission clusters formed by 19 (19.3%) of 82 Chinese IDUs (X^2^ = 16.50, *p*<0.01) (Table 4). Similarly, 20–100% NS5B sequences from Burmese IDUs formed transmission clusters dependent on different subtypes. A total of 10.5 transmission clusters were formed by 30 (41.7%) of 72 NS5B sequences from Burmese IDUs. This number was three times more than 3.5 transmission clusters formed by 7 (9.5%) of 74 NS5B sequences from Chinese IDUs (X^2^ = 20.01, *p*<0.01) (Table 4). There was no significant difference in the distribution of HCV transmission clusters between different HCV subtypes (*p*>0.05, except C/E2 sequences from Chinese IDUs with *p* = 0.027). Interestingly, all strains for the potential new HCV subtype existed in a same transmission cluster.

**Table 4:** Distribution of HCV transmission clusters in different subtypes and populations.

Genomic fragment	Nationality	Numbers of sequence and cluster	1a	1b	3a	3b	6n	6u	Potential new HCV subtype	Total
C/E2	Burmese IDUs (n = 92)	Total sequence	10	1	12	40	14	11	4	92 †
		Formed cluster	2	NA *	3	8	2.5‡	3	1	19.5
		Sequences within clusters	6	NA	7	20	6	6	4	49
		Percentage of clustered sequence (%)	60	NA	58.3	50.0	42.9	54.5	100.0	53.3
	Chinese IDUs (n = 83)	Total sequence	7	1	9	42	15	8	0	82
		Formed cluster	0	NA	0	6	2.5‡	0	NA	8.5
		Sequences within clusters	0	NA	0	13	6	0	NA	19
		Percentage of clustered sequence (%)	0.0	NA	0.0	30.9	40.0	0.0	NA	23.2
NS5B	Burmese IDUs (n = 72)	Total sequence	8	1	7	32	10	10	4	72 †
		Formed cluster	1	NA	0	6	2	1	0.5	10.5
		Sequences within clusters	4	NA	0	15	5	2	4	30
		Percentage of clustered sequence (%)	50.0	NA	0.0	46.9	50.0	20.0	100.0	41.7
	Chinese IDUs (n = 75)	Total sequence	6	1	9	37	10	10	1	74
		Formed cluster	0	NA	0	2	0	1	0.5	3.5
		Sequences within clusters	0	NA	0	4	0	2	1	7
		Percentage of clustered sequence (%)	0.0	NA	0.0	10.8	0.0	20.0	100.0	9.5

* NA: Not Applicable. One sequence was impossible to form a transmission cluster.

^†^ 09CNYNYJ-0508 was excluded from this table because of only one sequence available.

^‡^ If a transmission cluster contains both Burmese and Chinese IDUs, it was assigned as 0.5 for each population. The numbers of the transmission clusters were counted based on ML trees.

### Comparison of HCV subtype distributions among IDUs between Dehong and other regions of China

**Fig 3:**
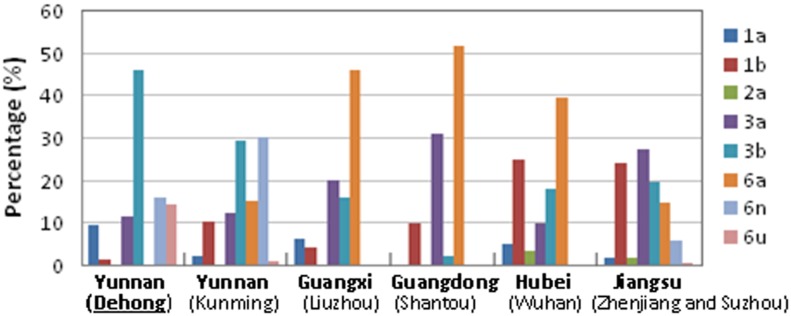
Comparison of HCV subtype distributions among IDUs between Dehong and other regions of China.

To investigate whether Dehong had a distinct genotype characteristic of HCV among IDUs, we compared the HCV subtype distributions among IDUs between Dehong and other regions in China, including Jiangsu [14,15], Guangdong [16], Guangxi [17], Hubei provinces [18] and Kunming city, the capital of Yunnan province [19]. HCV subtype distribution in Dehong was significantly different from those in other regions (*p*<0.05) (Fig 3). In Dehong, HCV subtype 3b was the absolutely predominant strains with a prevalence of 45.4% among IDUs, while subtypes 6a appeared to be the most predominant strains in Guangdong (51.5%, n = 136), Guangxi (46%, n = 96) and Hubei (39.3%, n = 61), and subtypes 3a (27%, n = 200) in Jiangsu. In Dehong, the second most common subtype was subtype 6n (15.7%), which was the most predominant strains in Kunming (30%, n = 100), and the third most common subtype was subtype 6u (13.5%), which was rarely found in other regions of China except Kunming city and Jiangsu province.

## Discussion

Currently, 4 HCV genotypes (1, 2, 3 and 6) and more than 10 subtypes (i.e. 1a, 1b, 2a, 3a, 3b, 6a, 6e, 6n, 6v, 6u) have been detected in China [9,20]. Subtypes 1b and 2a are transmitted mainly via blood transfusion, and were two of the most predominant subtypes with a nation-wide prevalence [20,21]. In recent years, IDU had become a major route of HCV infection and increasing HCV infection cases had been increasingly associated with IDU[8]. HCV prevalence among IDUs had increased rapidly, and remained at high prevalence rates especially at the worst-hit area by drugs (e.g. Yunnan and Guangxi) [11,12,17,22]. The distribution of HCV subtypes among IDUs was significantly different from that in general population [9]. Although HCV subtype 1b was the most common HCV subtype circulating among general Chinese population, it had very low prevalence among IDUs [8,9,14,15,17,19,23]. Genotypes 3 and 6 were the dominant prevalent subtypes among IDUs, and their prevalence were gradually extended to a wider geographic region [9,14,15]. Yunnan, a province of China which neighbors the biggest drug-producing areas the “Golden Triangle”, had severe HCV epidemics with very complex molecular epidemiological characterizations [19].

In the present study, we investigated the genetic diversity of HCV among IDUs in Dehong, a prefecture of Yunnan which borders with Myanmar. The cohort contains 118 Chinese and 141 Burmese IDUs. From the cohort, we detected 7 HCV subtypes, including 1a, 1b, 3a, 3b, 6n, and 6u, and a potential new HCV subtype. Subtype 3b (45.4%) was the absolutely predominant subtype in this cohort, followed by subtypes 6n (15.7%) and 6u (13.5%) (Table 1). Comparison showed that Dehong had a unique pattern of HCV subtype distribution among IDUs, significantly different from other regions of China (*p*<0.05), especially for genotype 6 (Fig 3). Subtypes 6n and 6u were the second and third top common strains in Dehong, but rarely found in other regions of China. In contrast, subtype 6a was the most predominant strains prevalent in Guangxi, Guangdong and Hubei, but not found in Dehong (Fig 3).

Genotype 6 was mainly circulating in Southeast Asia countries (e.g. Vietnam, Thailand, Myanmar, Laos, etc.), but rarely reported in other regions [24]. Many subtypes of genotype 6 were believed to originate in this area and to spread to the surrounding countries via cross-border transmission pattern [24]. As an important trade transfer station linking Southeast Asia and China, and the drug-trafficking channel linking “Golden triangle” and other regions of China and neighboring countries, Dehong may also play a crucial role in cross-border transmission of HCV as observed in HIV-1 [25,26]. Subtype 6u was first found from Myanmar in 2004, and characterized as a new HCV subtype by three nearly full-length genomic sequences from Dehong IDUs in 2008 [27,28], suggesting that subtype 6u originated in this region. In the following years, sporadic subtype 6u strains were identified in other regions of China [14,29]. The fact of high prevalence (14.1%) of subtype 6u in Dehong, together with few strains of this subtype reported in Myanmar, might suggest that subtype 6u originated in Dehong and further spread from here to Myanmar and other regions of China (Fig 3). Subtype 6n was the most predominant strains prevalent in Northern Myanmar (42–53%) [30]. The prevalence of subtype 6n in Northern Myanmar was substantially higher than those in Dehong (15.8%), Kunming (30%) and Thailand (22%) [19,31], suggesting a migration from Myanmar to Yunnan and other Southeast countries (e.g. Thailand). Sporadic detection of subtype 6n strains in other regions of China might suggest a further migration of 6n strains from Yunnan to other regions of China [14,15,29].

The China-Myanmar border area was the worst-hit region by HIV and HCV [11,12]. Previous studies showed that this region had very complex HIV-1 subtype diversity, including HIV-1 subtypes B, C, CRF01_AE, CRF02_AG, as well as a large number of HIV-1 inter-subtype recombinants [32,33]. Two HCV subtypes 6u and 6v had been first identified in Yunnan [27,34]. In this study, we detected six known HCV subtypes and found a potential new HCV subtype among IDUs in Dehong (Fig 2 and Table 3). Furthermore, we also found a strain 09CNYNYJ-0508 that did not cluster within any known subtype clade (Fig 2). Similarly, a previous strain DH027 in Dehong also did not cluster within any known subtype clade [35]. They might represent new unknown HCV subtypes that need to be determined in future. These suggested that Dehong had very complex HCV subtype diversity. Because the sample size in this study was not big enough, there might be some new unknown strains/subtypes that were still not detected and identified. On the other hand, we found 6 NS5B and 7 C/E2 sequences highly similar to the potential HCV new subtype using HCV BLAST. Phylogenetic analyses showed that they formed a well supported clade with a bootstrap value of 100% together with the sequences obtained in this study (S1 Fig). Because these sequences were isolated in Dehong in 2014 [13], later than the sequences obtained during 2011–2012 in this study, it implies that the new HCV subtype was expanding among IDUs in Dehong. The appearance of this new subtype among IDUs could be important to public health efforts.

There was no significant difference in HCV subtype characterizations between Chinese and Burmese IDUs (*p* = 0.667) (Table 2). Because the Burmese IDUs enrolled in this study were living in Dehong or stayed here in a relatively long period of time, they might acquire HCV infection here, which provides an explanation for the consistence in HCV genetic diversities between Chinese and Burmese IDUs. In addition, if both sides of the border had consistent HCV genetic diversities, it can also explain this consistence. However, because the subtype characterization of HCV in the Burmese side of the border was unavailable, we did not easily make a conclusion.

On the other hand, we found that about half (41.7–53.3%) of HCV sequences from Burmese IDUs formed small transmission clusters, significantly higher than that (9.5–23.2%) from Chinese IDUs (*p*<0.01) (Table 4). Among these transmission clusters, only 4.5% (1/28 in C/E2 fragment) to 7.1% (1/14 in NS5B fragment) of clusters contain the sequences from both Chinese and Burmese IDUs (Fig 2). These suggested that the Chinese and Burmese IDUs were relatively isolated from each other in IDU behavior and the Burmese IDUs might prefer to inject drugs themselves together. These differences might be due to different cultural backgrounds between Chinese and Burmese. However, the separation would eventually be broken when these Burmese are gradually integrated into the local culture and life. By then, HCV transmission and mixing of various HCV subtypes between Chinese and Burmese IDUs will become more common and severe.

## Conclusions

The present study described the genetic diversity of HCV among IDUs in Dehong, Yunnan, China. Six HCV subtypes, including 1a, 1b, 3a, 3b, 6n and 6u, were detected, and a potential new subtype was reported among the IDUs in Dehong. Dehong had a unique pattern of HCV subtype distribution, with a relatively higher prevalence of HCV subtype 6u than other regions of China. The unique genotype distribution and complex diversity of genotype 6 may be related to the special geographical position of Dehong. In addition, more HCV sequences from Burmese IDUs formed small transmission clusters than that from Chinese IDUs (*p*<0.01), and few HCV sequences from Burmese IDUs clustered with those from Chinese IDUs, suggesting that the Burmese IDUs might prefer to inject drugs themselves together. Since the sample size of Dehong IDUs is relatively small and the related information of Northern Myanmar is unavailable, this study is unable to provide the most comprehensive scenario about HCV transmission between Chinese and Burmese IDUs.

## Materials and Methods

### Ethics statement

This study was done according to the Helsinki II Declaration and was approved by the Ethics committee of Kunming Institute of Zoology, Chinese Academy of Sciences. All the 259 participants were voluntary in the present study and provided written informed consents before participating in this study.

### Study participants and sample collection

Under the support of HIV/AIDS Asia Regional Program Yunnan Management Office and local government, a cross-sectional survey was conducted with IDUs from urban detoxification centers or communities in Dehong prefectures of Yunnan province during 2009 to 2012. Socio-demographic characteristics including gender, age, occupation, ethnicity, marital status, and education level was collected by anonymous questionnaire-based interviews. Whole blood samples (about 5 mL) were collected from each participant using sterile ethylenediaminetetraacetic acid (EDTA) tubes. Plasma was separated by centrifugation. HCV sero-positive was determined using diagnostic enzyme-linked immunosorbent assay (ELISA) kit (Shanghai Kehua Bio-engineering Co., LTD). The samples from 259 IDUs, including 118 Chinese and 141 Burmese, were tested as HCV sero-positive and subjected to subsequent molecular epidemiological analyses.

### RNA extraction and reverse transcription polymerase chain reaction (RT-PCR)

Total RNA was extracted from 140μL of HCV-positive plasma with the QIAamp^®^ Viral RNA Mini Kit (QIAGEN^®^). Multiple RT-nested PCR was performed to amplify two HCV genomic fragments C/E2 and NS5B as described previously [14]. In brief, reverse primers C/E2-R1 and NS5B-R1 were used in the RT reaction to obtain two cDNA fragments. The cDNA products were used as the templates for the amplification of C/E2 and NS5B genomic fragments with the nested PCR. After the identification by 1% agarose gel, the PCR products were sent to Shanghai Biosune Biotech Co. ltd (Shanghai, China) for sequencing. The primer sequences are shown in S2 Table.

### HCV genotyping and phylogenetic analyses

The sequences of all samples were aligned together with HCV subtype reference sequences downloaded from HCV database using the MUSCLE program implemented in MEGA 5.0. The best substitution model for maximum likelihood (ML) analysis was estimated using Jmodeltest v2.1.4. The “GTR+I+G” was the best substitution model for both C/E2 and NS5B sequence sets. The phylogenetic trees of C/E2 and NS5B were constructed using MEGA 5.0 program with the neighbor-joining (NJ) method under the Tajima-Nei model and ML method under the GTR+I+G model. The reliabilities of tree topologies were evaluated by the bootstrap method with 1000 replications. The genetic distance was computed using “Distance” program in MEGA 5.0. Transmission clusters were determined based on the strong statistical support of high bootstrap values (>95%) and short genetic distances (<4.5%) in both ML and NJ trees [36]. If a transmission cluster contains sequences from both Burmese and Chinese IDUs, it was assigned as 0.5 for each population.

### Statistical analysis

Statistical analyses were performed by the SPSS software (version 17.0; SPSS, Inc., Chicago, IL, USA). Statistical comparisons of HCV subtype distributions between Dehong and other regions of China, and formation of transmission clusters between different subtypes were performed by the chi-square (χ^2^) test. A probability (*p*) value < 0.05 was considered statistically significant.

## Supporting Information

S1 Fig ML subtrees of C/E2 (A) and NS5B (B) fragments of the potential new HCV subtype using the sequences from previous studies and this study. The previous sequences were highlighted by red circles (Ref. [13]). All sequences of the potential new HCV subtype formed a large clade.
(DOC)

S1 Table Social-demographic characteristics of HCV infected IDUs in Dehong. (DOC)

S2 Table Information of the primer pairs used in this study. (DOC)
