# Supplementary material for: HCV Diversity among Chinese and Burmese IDUs in Dehong, Yunnan, China
Source: PLoS One. 2016 Sep 22;11(9):e0163062. doi: 10.1371/journal.pone.0163062 (PMC5033387; doi:10.1371/journal.pone.0163062)
Supplement: S1 Fig — The previous sequences were highlighted by red circles (Ref. [13]). All sequences of the potential new HCV subtype formed a large clade. (DOC) [file pone.0163062.s001.doc]

**S1 Fig. ML subtrees of C/E2 (A) and NS5B (B) fragments of the potential new HCV subtype using the sequences from previous studies and this study.** The previous sequences were retrieved from and highlighted by red circles. All sequences of the potential new HCV subtype formed a large transmission cluster.
