# Supplementary material for: HCV Diversity among Chinese and Burmese IDUs in Dehong, Yunnan, China
Source: PLoS One. 2016 Sep 22;11(9):e0163062. doi: 10.1371/journal.pone.0163062 (PMC5033387; doi:10.1371/journal.pone.0163062)
Supplement: S1 Table — (DOC) [file pone.0163062.s002.doc]

**S1 Table.** Social-demographic characteristics of HCV infected IDUs in Dehong.

|  | **Chinese**  (n=118) | **Burmese**  (n=141) | **Total (%)** |
| --- | --- | --- | --- |
| Occupation | | | |
| Farmer | 93 | 116 | 209 (88.2) |
| Worker | 14 | 8 | 22 (9.3) |
| Unemployed | 1 | 5 | 6 (2.5) |
| Ethnic background | | | |
| Han | 48 | 30 | 78 (31.1) |
| Dai | 45 | 34 | 79 (31.5) |
| Jingpo | 14 | 53 | 67 (26.7) |
| Other | 5 | 22 | 27 (10.8) |
| Marriage status | | | |
| Single | 53 | 63 | 116 (45.8) |
| Married | 32 | 71 | 103 (40.7) |
| Divorced | 9 | 2 | 11 (4.3) |
| Unmarried cohabitation | 19 | 3 | 22 (8.7) |
| Educational status | | | |
| Illiterate | 15 | 28 | 43 (17.2) |
| Primary school | 49 | 61 | 110 (44.0) |
| Junior high school | 43 | 31 | 74 (29.6) |
| Senior high school | 5 | 12 | 17 (6.8) |
| College or above | 1 | 5 | 6 (2.4) |
| Age (years old) | | | |
| <20 | 1 | 1 | 2 (0.8) |
| 20-25 | 3 | 10 | 13 (5.1) |
| 26-30 | 29 | 29 | 58 (22.9) |
| 31-35 | 34 | 38 | 72 (28.5) |
| 36-40 | 26 | 28 | 54 (21.3) |
| 41-45 | 13 | 14 | 27 (10.7) |
| >45 | 7 | 20 | 27 (10.7) |
