# Supplementary material for: HCV Diversity among Chinese and Burmese IDUs in Dehong, Yunnan, China
Source: PLoS One. 2016 Sep 22;11(9):e0163062. doi: 10.1371/journal.pone.0163062 (PMC5033387; doi:10.1371/journal.pone.0163062)
Supplement: S2 Table — (DOC) [file pone.0163062.s003.doc]

**S2 Table. Information of the primer pairs used in this study**.

| **Region** | **Nested PCR** | **Name** | **Sequences（5’-3’）** | **Position in H77 genome** | **Size (bp)** |
| --- | --- | --- | --- | --- | --- |
| **C/E2** | 1^st^ PCR | C/E2-F1 | GCCGACCTCATGGGGTACAT | 732-751 | 1480 |
|  |  | C/E2-R1 | ARTTBTYDGTRCANGGRTARTGCCA | 2187-2211 |  |
|  | 2^nd^ PCR | C/E2-F2 | CCYGGTTGCTCYTTYTCTATCTT | 849-871 | 1303 |
|  |  | C/E2-R2 | GTNADCCANGGNCCNGMNCCRCA | 2130-2152 |  |
| **NS5B** | 1^st^ PCR | NS5B-F1 | GGSTTYTCNTATGAYACCMGVTGYTTTGA | 8247-8275 | 1104 |
|  |  | NS5B-R1 | CTACCCCTACNGHDAGTAGGAGTAGGC | 9325-9351 |  |
|  | 2^nd^ PCR | NS5B-F2 | GCTGYTTTGAYTCAACNGTCAC | 8266-8287 | 1036 |
|  |  | NS5B-R2 | GRGCHYGVGACACGCTGTGATANATGTC | 9276-9303 |  |
